# Preparation of Colloidal Gold Immunochromatographic Strip for Detection of Paragonimiasis skrjabini

**DOI:** 10.1371/journal.pone.0092034

**Published:** 2014-03-18

**Authors:** Ying Wang, Lifang Wang, Jianwei Zhang, Guangxi Wang, Wenbi Chen, Lin Chen, Xilin Zhang

**Affiliations:** 1 Institute of Tropical Medicine, Third Military Medical University, Chongqing, China; 2 Department of Pathogenic Biology, Luzhou Medical College, Sichuan, China; 3 Department of Physics, Chengdu Medical College, Sichuan, China; 4 Department of Pathogenic Biology, Third Military Medical University, Chongqing, China; New York University, United States of America

## Abstract

**Background:**

Paragonimiasis is a food-borne trematodiasis, a serious public health issue and a neglected tropical disease. *Paragonimus skrjabini* is a unique species found in China. Unlike paragonimiasis westermani, it is nearly impossible to make a definitive diagnosis for paragonimiasis skrjabini by finding eggs in sputum or feces. Immunodiagnosis is the best choice to detect paragonimiasis skrjabini. There is an urgent need to develop a novel, rapid and simple immunoassay for large-scale screening patients in endemic areas.

**Methodology/Principal Findings:**

To develop a rapid, simple immunodiagnostic assay for paragonimiasis, rabbit anti-human IgG was conjugated to colloidal gold particles and used to detect antibodies in the sera of paragonimiasis patients. The synthesis and identification of colloidal gold particles and antibody-colloidal gold conjugates were performed. The size of colloidal gold particles was examined using a transmission electron microscope (TEM). The average diameter of colloidal gold particles was 17.46 nm with a range of 14.32–21.80 nm according to the TEM images. The formation of antibody-colloidal gold conjugates was monitored by UV/Vis spectroscopy. Excretory-secretory (ES) antigen of *Paragonimus skrjabini* was coated on nitrocellulose membrane as the capture line. Recombinant *Staphylococcus* protein A was used to prepare the control line. This rapid gold immunochromatographic strip was assembled in regular sequence through different accessories sticked on PVC board. The relative sensitivity and specificity of the strip was 94.4% (51/54) and 94.1% (32/34) respectively using ELISA as the standard method. Its stability and reproducibility were quite excellent after storage of the strip at 4°C for 6 months.

**Conclusions/Significance:**

Immunochromatographic strip prepared in this study can be used in a rapid one-step immunochromatographic assay, which is instantaneous and convenient.

## Introduction

Paragonimiaisis is a prevalent food-borne zoonosis caused by *Paragonimus* and is found in African, South American and Asian countries such as China, Japan, Liberia, Nigeria and Venezuela [1], [2]. More than 50 species have been described all over the world including 38 species in China, among which the most predominant infections are *Paragonimus westermani* and *Paragonimus skrjabini* [3]. *P. westermani* mainly causes pulmonary pargonimiasis and sometimes causes abdomen, lymph nodes or brain paragonimiasis. *P. skrjabini* is a unique species only found in China. In humans, few *P. skrjabini* can develop into adults in the human lung. Most of the parasites are in the juvenile stage and can migrate into many different organs including muscles, subcutaneous tissues and even the brain [4]. As a result, paragonimiasis skrjabini of humans usually manifests as complex clinical symptoms, which often resulted in misdiagnosis and delayed treatment. Definitive diagnosis of human paragonimiasis is mainly based on the finding of characteristic eggs in sputum or feces. However, finding eggs is not easy in mild, latent, ectopic or chemotherapeutically-affected cases, especially for *P. skrjabini* infected cases. Therefore, several immunologic tests have been developed as diagnostic tools for paragonimiasis. Previously, the commonly used tests for paragonimiasis skrjabini were immunodiagnostic methods to detect specific antibodies or antigens using intradermal test (IDT) or Enzyme-linked immunosorbent assay (ELISA) [5], [6]. Due to hypersensitivity reactions and low specificity and sensitivity [7], [8], IDT is now rarely used in clinic. ELISA is widely used now for its high specificity and good sensitivity [8]. However, ELISA is not quick and not easy enough for the primary hospitals and field studies, since it requires special equipments and reagents, and takes several hours. There remains a need to develop a novel, rapid and simple immunoassay, especially for screening patients on a large scale in endemic areas.

Immunochromatographic strip (ICS) is a rapid one-step immunochromatographic assay [9]. The convective mass transfer of the immunoreactant to the binding partner allowed the assay to be performed with no handling of reagents [10]. It is an instantaneous and convenient examination [11]. Here we used purified rabbit anti-human IgG conjugated with colloidal gold to detect antibodies in the sera of paragonimiasis patients. This assay was found to be rapid, simple, cheap and effective for detection of paragonimiaisis in the endemic areas.

## Materials and Methods

### Ethics statement

Oral informed consent was obtained from all adult participants or parents/guardians of minors enrolled in this study. As many participants coming from remote rural area were illiteracy, written consent was not easy to be obtained. All adult participants provided their own oral consent to agree to participate. And, all minors had oral consent given from a parent/guardian before participation in the study. They were told that their sera would be used to study the immunodiagnosis methods, and their private information would be safe. And, the agreement from the participants was recorded on a uniform sheet with their fingerprints. This study, including using oral consent, was approved by the Ethics Committee for Health Research, Third Military Medical University, China. Approval for collecting the crabs used in this study was obtained from provincial and district health authorities and village leaders. The animal care and use protocol was approved by the Institutional Animal Care and Use Committee of the Third Military Medical University, China (SYXK-PLA-2007035). The experiments conformed to the Guideline on the Humane Treatment of Laboratory Animals stipulated by the Ministry of Science and Technology of the People's Republic of China (MOST).

### Materials

#### Excretory-secretory (ES) antigen

Freshwater crabs were collected from Xianfeng Town, Xingwen County in Sichuan Province. Five Wistar rats were infected orally with the isolated *Paragonimus skrjabini* metacercaria (50 metacercariae per rat) which were identified morphologically [4]. Thirty-five days later, the rats were sacrificed to collect adolescent worms from the peritoneal cavity. Parasites were incubated in sterile saline at room temperature for 12 h. The medium was collected by centrifuging (1700 rpm) for 30 min. Then, the ES supernatant was transferred to a dialysis bag and embedded in solid PEG 6000 to concentrate the ES protein. Finally, the concentration of worm ES protein was determined by ultraviolet spectroscopy. As a result, the final concentration was 5.08 mg/ml. The dialysis concentrated worm ES protein was preserved at −20°C until use.

#### Test sera

Fifty-four sera samples were obtained from patients with paragonimiaisis skrjabini who had eaten raw fresh water crabs and had typical clinical manifestations such as subcutaneous mass type, pleurisy type, cerebral type, pericarditis type and abdominal type. Positive sera were detected by ELISA and the patients were successfully treated by praziquantel. Serum samples of schistosomiasis and clonorchiasis patients diagnosed by pathogen detection were used as the control, which were obtained from Jiangsu Institute of Parasitic Diseases, China. Sera from thirty-four healthy people were provided by volunteers from Chongqing City.

Rabbit antihuman IgG and recombinant *Staphylococcus* protein A were obtained from Beijing Biosynthesis Biotechnology Company. The protein (1 mg/ml) was reconstituted with 1 ml of 0.01 M Phosphate-buffered saline (PBS, pH 7.4). Nitrocellulose membrane with backing (M135s), glass fiber, and absorption pad were purchased from Millipore (USA). Chloroauric acid (Gold chloride) (AuCl_3_·HCl·4H_2_O), sodium citrate (Na_3_C_6_H_5_O_7_·2H_2_O), Polyethylene glycol (PEG) 20000 were purchased from the Sinopharm Chemical Reagent company (China). Deionized and distilled water was used in this experiment. Phosphate-buffered saline (PBS, pH 7.4, 0.01 M in 0.85% NaCl) was prepared. All other chemicals used in the present study were either analytical pure or with highest quality.

### Synthesis and characterization of colloidal gold

Colloidal gold particles with a mean diameter of 17.46 nm were prepared according to the method of Frens [12]. An aqueous solution of chloroauric acid (100 ml 0.01% (W/V) AuCl_3_·HCl·4H_2_O) was heated to boiling point, followed by adding 2 ml of 1.0% (W/V) sodium citrate solution. The reaction solution was stirred simultaneously and gently boiled for 5 min until the color of the solution turned from straw yellow into black and eventually red. The obtained colloidal gold solution can be stored at 4°Cfor several months and it was used for conjugation with purified antibody. Colloidal gold particles were examined by a transmission electron microscope (TEM) performed on a TECNAI-10 (Philips Co., The Netherlands) and operated at an acceleration voltage of 80 kV with a magnification of 135,000. The sample was prepared by placing a drop of colloidal gold on a carbon-coated TEM copper grid. The film was allowed to dry for 10 min, and the excess solution was removed using a tissue paper. The size distribution of the colloidal gold particles from photographs enlarged three times was measured, and the TEM images were selected at least 100 counts.

### Optimization and formation of antibody-colloidal gold conjugates

Antibody solution (rabbit antihuman IgG, 1 mg/ml, 3 μl) was added to 100 μl of colloidal gold solution with pH values varying from 5–9 in triplicate. Five minutes later, 20 μl of 10% NaCl was added. Two hours later, the minimum pH keeping the red color without change was considered as the optimum pH. Then, various amount of purified antibody solution (0.1 mg/ml, from 2 to 20 μl) was added to wells with 100 μl of colloidal gold solution at the optimum pH. After the addition of 20 μl of 10% NaCl, color change was observed 2 hours later and the minimum amount of antibody for stabilizing colloidal gold particles was determined.

According to the above results, appropriate amount of purified rabbit antihuman IgG was added drop-wise to colloidal gold solution with optimum pH. The mixture was stored for 2 hours at 4°C and centrifuged at 10000 rpm for 1 hour. The supernatant with unconjugated antibody was carefully removed and the resulting pellet re-suspended in 4 ml of incubation buffer (0.01 M PBS) containing 2% BSA. And, 5 ml of 1% Polyethylene glycol-20000 (PEG-20000) was added to block the unreacted sites on the gold colloids, which was stored at 4°C for further experiments. The formation of antibody-colloidal gold conjugation was monitored with UV/Vis spectroscopy (Ultropec 2100 pro UV, Amersham Pharmacia, Sydney).

### Preparation of immnunochromatographic strip

Immnunochromatographic test strip was constructed using the method of Paek et al. [11]. Gold-labeled antibody probe (without dilution) was jetted onto the glass fiber and dried at room temperature. Recombinant *Staphylococcus* protein A (1 mg/ml) and ES antigen (5 mg/ml) in PBS were jet-positioned onto a nitrocellulose membrane as two discrete zones, one for control and another for test. The remaining active sites on the membrane were blocked by incubation with 5% nonfat milk in PBS overnight at 4°C. The membrane was washed three times with PBS and then air dried. The sample application pad, antibody-gold conjugated pad, nitrocellulose membrane and the absorption pad were air dried and laminated successively. The laminated sheet was then cut into individual strips (3 mm/strip) with a pair of small medicine scissors.

### Procedure of serum samples detection

For each test, 5 μl of solution of colloidal gold conjugated with rabbit antihuman IgG was first added on the sample pad. Then, the strip was immersed in the diluted serum samples (15 μl sera+100 μl PBS), allowing the sample to migrate upward. After 10 minutes, an image containing the color signal was produced in the strip and the results were judged by the color of the test and control lines. If both the detection band and the control band turned red, the sample was recorded as positive. If the control band turned red but the detection band was not colored, it was recorded as negative. If neither band was colored, the test reagents were assumed to be not working.

## Results

### Characterization of the colloidal gold particle

**Figure 1:**
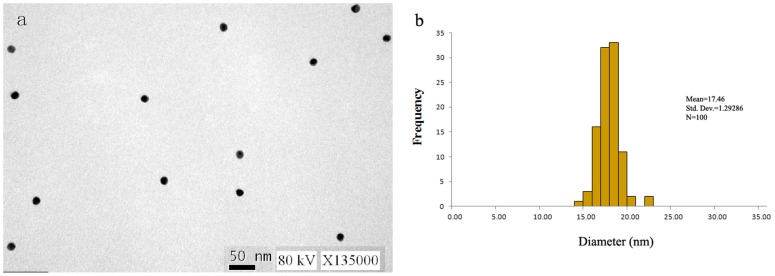
(a) The TEM images of gold nanoparticle and (b) their size distribution. Image reveals particles of varying size and shape with an average particle diameter of 17.46

Colloidal gold particles were synthesized by chemical method using the reduction of chloroauric acid (AuCl_3_·HCl·4H_2_O). Chloroauric acid was reduced to gold atoms by sodium citrate and many of the gold atoms accumulated into colloidal gold solution. The TEM image showed well-dispersed colloidal gold particles (Fig. 1a). The average diameter of the colloidal gold particles was 17.46 nm (Fig. 1b). TEM image indicated that the colloid gold particles were almost of the same diameter with a range of 14.32–21.80 nm (Table S1), which provided a good basis for preparation of ICS.

### Conjugation optimization and characterization of antibody-gold conjugates

To stabilize colloidal gold particles, the optimum pH of antibody adsorption was determined to be 7.6. At this pH, 0.01 mg/ml antibody was confirmed to be the minimum amount for stabilizing colloidal gold solution. To ensure that enough antibody was used to conjugate with the gold particles and stabilize the colloidal gold, 0.011 mg/ml was determined to be the optimum concentration of purified antibody for the conjugation.

**Figure 2:**
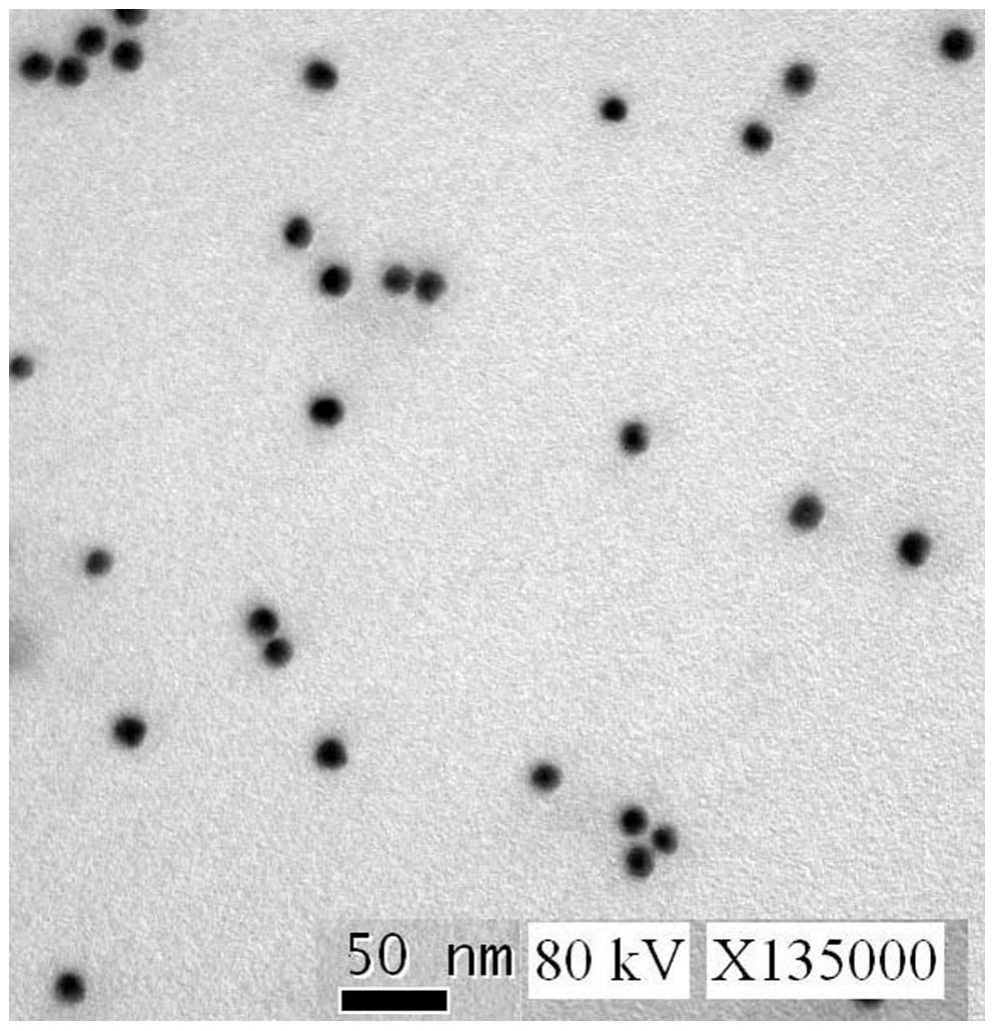
The TEM images of antibody-gold conjugates.

**Figure 3:**
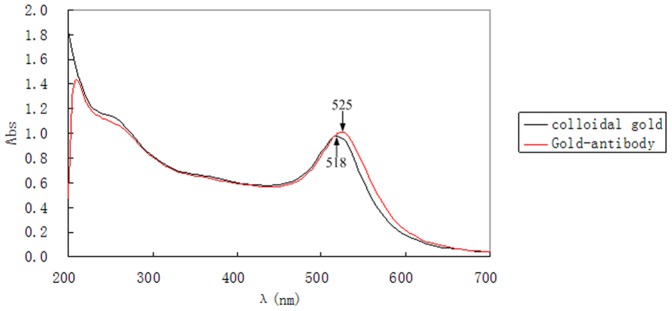
UV/Vis spectra of colloidal gold and the antibody-gold conjugate. Black line: colloidal gold solution; red line: the antibody-colloidal gold conjugate.

According to the TEM image, the colloidal gold particles were surrounded by a halo with low electron density after absorption of the antibody although without negative staining (Fig. 2). According to the UV/Vis spectra of the colloidal gold and conjugates, there was a shift of the peaks by antibody treatment. The peak at 518 nm of the colloidal gold curve was due to the surface resonance of colloidal gold particles. Added with the antibody, the surface resonance band shifted a little (Fig. 3). The band shift from 518 nm to 525 nm observed in the plasmon resonance was caused by the interaction of the antibody and colloidal gold particles [13], [14].

### Sensitivity, specificity and cross reactivity of the strip

**Figure 4:**
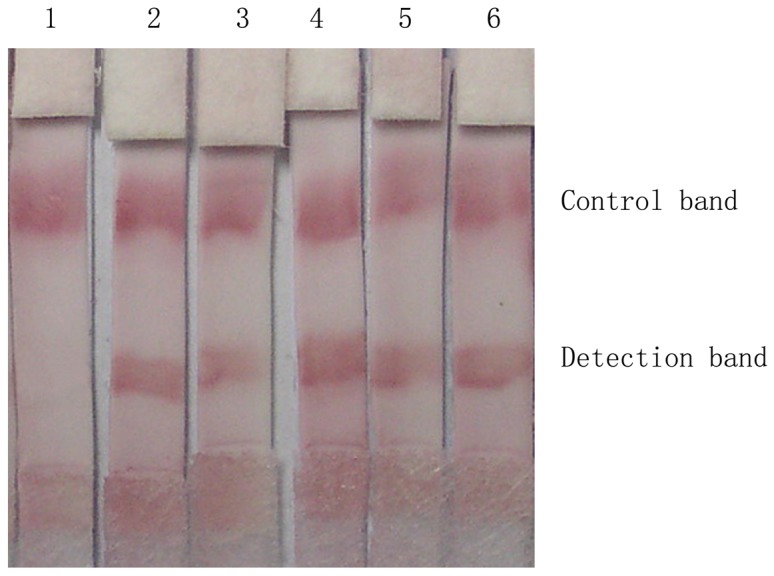
Examples of positive and negative tests for paragonimiasis using immunochromatographic strip (ICS). Lane 1: Negative result. Lanes 2,3,4,5,6: Positive.

As shown in Fig. 4, the sample was recorded as positive if two clear red lines were observed. If the control band turned red but the detection band was not colored, it was recorded as negative. The sensitivity, specificity and cross reactivity of the strip was evaluated in comparison to other sera of trematodiasis (Table 1). Our results showed that the sensitivity was 94.44% and the specificity was 94.12% for paragonimiasis. Cross-reactions with schistosomiasis and clonorchiasis were 4% (1/25) and 6.67% (1/15), respectively. The colloidal gold immnunochromatographic strip result was compared with that from ELISA (Table 1). There was no significant difference in sensitivity (χ2  = 3.09, P = 0.079) and specificity (χ2  = 2.06, P = 0.151) between the two assays. Youden's index of the ICS was 89% for paragonimiasis.

**Table 1:** Sensitivity and specificity investigation and comparison of colloidal gold Immunochromatographic strip and ELISA using *Paragonimus skjabini* ES protein as antigens.

Group	Number	ELISA			immunochromatographic Strips		
		Positive (No.)	Negative (No.)	Positive rate (%)	Positive (No.)	Negative (No.)	Positive rate (%)
Paragonimiasis	54	54	0	100	51	3	94.4
Healthy people	34	0	34	0	2	32	5.9
Schistosomiasis	25	Not done	Not done	-	1	24	4.0
Clonorchiasis	15	Not done	Not done	-	1	14	6.7

The schistosomiasis and clonorchiasis cases were confirmed by pathogen detection. And,the sera were obtained from Jiangsu Institute of Parasitic Diseases, China.

### Stability of the strip

After storage at room temperature for at least 6 months, the ICS was still effective. Ten stored strips were used to test the positive paragonimiasis sera. Another ten stored strips were used to test the healthy sera. All the test of paragonimiasis sera showed obviously positive result. And, all the test of healthy sera showed negative result. On the same batch, we also randomly selected three test strips used for repeated tests. The results were similar, which indicated that the strips were stable with little variability after storage for six months at room temperature.

## Discussion

Paragonimiasis is mainly prevalent in tropical Asia such as China, India [15], Lao PDR [16] and Vietnam [17]. In addition, people outside of Asia may also suffer from this disease [18]. The World Health Organization(WHO) estimates that about 20.7 million people are suffering from paragonimiasis [1]. Paragonimiasis is a serious public health issue in the region surrounding the Three Gorges Reservoir, China, where *P. skrjabini* has been identified as the most significant agent of human paragonimiasis in this area [4]. Extrapulmonary paragonimiasis is due to aberrant migration of juvenile worms, which usually results in diverse and complex clinical symptoms and misdiagnosis. Therefore, immunodiagnosis is commonly used in this disease because it is hard to find the eggs of *P. skrjabini* from patients' sputum or excreta. A quick and easy immunodiagnosis method with high specificity and sensitivity is in urgent need. Immunochromatographic test is a good choice and has been widely used as a kind of rapid diagnostic test for various parasitic diseases such as malaria, schistosomiasis, etc. [19]–[21].

In the present study, we used colloidal gold as a reporter, which has been widely used [22]. The adsorption of proteins to gold particles is by a non-covalent process based on three separate but dependent phenomena: ionic interaction between the negatively charged nanoparticle and the positively charged sites on the protein, hydrophobic attraction between the protein and the metal surface, and dative bonding between the metal and the conducting electrons of nitrogen and sulphur atoms of the protein [23]. The performance of bio-nanoparticles in immunoassays depends on both the chemical properties of the gold particles as well as the bioactivity of the protein. Preparation of high-quality colloid gold solution is a key step to ensure the peculiarity and sensibility of the test strip. Different sized gold nanoparticles have different conjugation efficiencies under different pH values and concentrations of antibody [24]. The smaller gold particles have the less requirement for antibody concentrations to avoid aggregation [24]. Our findings indicate that the optimum pH is at 7.6 and the minimum amount of antibody is 0.01 mg/ml. TEM image indicates that the colloid gold particles were almost of the same diameter and aggregated free. Other researchers usually characterized gold particles with bound antibody by TEM using a contrasting agent such as uranyl acetate or digital contrast enhancement [23], [25]–[27]. In the present study, the difference between gold particles before and after absorption of antibody was obvious in the TEM figures although without contrasting agent or technique. Besides, upon addition of antibody λ_max_ shifted from 518 nm to 525 nm indicating good protein conjunction.

The specificity and sensitivity of ICS is also dependent on the antigen used in the test strip. ES purified antigen was used as detection line in this study. ES antigens were products emanated from the intestine of adult worms, as well as uterine contents which female worms released along with transmission stage eggs or larvae. ES can interfere with every aspect of host immunity from initial recognition to end-stage effector mechanisms [28]. ES becomes antigenic to the host by being continuously released outside the fluke and is the main antigen-antibody reaction stimulating factor. As such, ES antigen is better than crude antigen in immunodiagnosis [29]. *Staphylococcus* protein A can combine with most of the immunoglobulin Fc segment, so it was often used as a broad-spectrum secondary antibody by strong binding with IgG. In this study, staphylococcal protein A was used to prepare the control line in the strip.

According to the results of this study, there was no significant difference in sensitivity and specificity between ICS and ELISA. However, ICS has many advantages compared to ELISA, such as needing no equipment or trained operators and having easily readable results in 15 min. The test strip has a potential for quantitative monitoring and can be manufactured commercially [30]. This will help to screen suspected patients in endemic areas, differential diagnosis in primary hospitals and assessment of the treatment efficacy. However, it's still unknown if the strip is species specific. ES antigens were used in this study to prepare ICS. ES antigens are good candidates for the immunological diagnosis, but are difficult to develop in large scale. Therefore we shall obtain the recombinant ES antigens with the recombinant DNA technique, which will be good for the development of ICS in large quantity with high quality to increase the species specificity.

## Acknowledgments

We acknowledge the volunteers for providing their sera in this study. We thank Professor Liwang Cui from Pennsylvania State University and Steve Shi from Irvine, California for polishing the English expressing.

## Supporting Information

Table S1 **The diameters of the gold particles.** They were measured using the Measure Tool of Photoshop software based on the photos taken by TEM.
(XLSX)
